# Antioxidant Mechanisms and ROS-Related MicroRNAs in Cancer Stem Cells

**DOI:** 10.1155/2015/425708

**Published:** 2015-04-29

**Authors:** Ilaria Dando, Marco Cordani, Elisa Dalla Pozza, Giulia Biondani, Massimo Donadelli, Marta Palmieri

**Affiliations:** Department of Life and Reproduction Sciences, Biochemistry Section, University of Verona, Strada Le Grazie 8, 37134 Verona, Italy

## Abstract

Increasing evidence indicates that most of the tumors are sustained by a distinct population of cancer stem cells (CSCs), which are responsible for growth, metastasis, invasion, and recurrence. CSCs are typically characterized by self-renewal, the key biological process allowing continuous tumor proliferation, as well as by differentiation potential, which leads to the formation of the bulk of the tumor mass. CSCs have several advantages over the differentiated cancer cell populations, including the resistance to radio- and chemotherapy, and their gene-expression programs have been shown to correlate with poor clinical outcome, further supporting the relevance of stemness properties in cancer. The observation that CSCs possess enhanced mechanisms of protection from reactive oxygen species (ROS) induced stress and a different metabolism from the differentiated part of the tumor has paved the way to develop drugs targeting CSC specific signaling. In this review, we describe the role of ROS and of ROS-related microRNAs in the establishment and maintenance of self-renewal and differentiation capacities of CSCs.

## 1. Introduction

Historically, cancer cells were considered derived from a single tumorigenic clone that originates from genetic alterations, including mutations of oncogenes and tumor suppressor genes. However, the observation that cancer tissues exhibit significant heterogeneity in several features, including morphology, cell surface antigens, and gene expression, led to the idea that cancer may constitute a cellular hierarchy with cancer stem cells (CSCs) at the apex, as in normal tissue development [1]. In this model, epigenetic changes and signaling events regulate the structural organization of the tumor during its differentiation phases. Recent advances in sequencing technologies have clarified that tumors do not have a single genome but instead comprise multiple genomes belonging to distinct subclones. Thus, although often considered as mutually exclusive models to describe tumorigenesis, the genetic and hierarchical models may now be viewed as integrated processes in which stemness represents a central biological property of cancers on which many driver mutations take place [1]. An additional facet to this already complex picture has been added by a growing number of reports showing the remarkable degree of plasticity of the non-stem cell population, which challenges the idea of a unidirectional differentiation of cancer cells [2]. In 1994, Lapidot et al. made the first validation of the CSC hypothesis by isolating CSCs in acute myeloid leukemia [3]. After that, CSCs were identified in solid cancers and today they are continuously identified and isolated in a growing list of tumor types [2]. The cardinal property of a stem cell, whether normal or malignant, is self-renewal, which is the key biological process where, upon cell division, a stem cell produces one (asymmetric division) or two (symmetric division) daughter cells that retain the capacity for self-renewal. The asymmetric division leads also to terminally differentiated cells, with limited proliferative potential, that represent the bulk of the tumor mass. The CSCs have several molecular features determining survival advantages over the differentiated cancer cell populations, including the resistance to radio- and chemotherapy. Gene expression profiling has shown a correlation between poor clinical outcome and the presence of CSC features, further supporting the relevance of stemness properties in cancer, that may thus be considered strategic targets for cancer eradication [2]. Recently, a number of studies have demonstrated that the altered production of reactive oxygen species (ROS) in CSCs may represent possible targets for treatment of human neoplasia. In this review, we focus on the role of hypoxia, of ROS, and of antioxidant mechanisms, including ROS-related microRNAs, in the establishment and maintenance of self-renewal and differentiation capacities of CSCs.

## 2. Roles of Hypoxia and ROS in Cancer Stem Cell Biology

Poor or altered vascularization usually present in heterogeneously distributed areas within solid tumors determines hypoxic or anoxic zones. The low oxygen tension generally provides strong selective pressure for tumor growth and eventually favors survival of the most aggressive malignant cells [4]. Hypoxia within a neoplastic mass is considered an independent prognostic indicator of poor clinical outcome with a significant risk to develop metastasis and cancer progression [5, 6]. Under hypoxic conditions, the hypoxia inducible factor- (HIF-) 1*α*, which has been found overexpressed in many human cancers [7], is stabilized, dimerizes with HIF-1*β*, and translocates into the nuclei. At the promoter of hypoxia-dependent target genes, the binding of HIF-1 to a specific sequence, named hypoxia-responsive element (HRE), activates a complex genetic program for several cellular changes to efficiently counteract the decreased oxygen tension [8]. Indeed, HIF-1 activates transcription of genes involved in crucial features of cancer biology, such as angiogenesis, cell survival, glucose metabolism, and invasiveness, representing a target for a selective cancer therapy [9, 10]. Noteworthy, several genes associated with the hypoxic response in normal cells, such as Glut1, Serpin B9, and VEGF, are upregulated in CSCs [10, 11]. Furthermore, hypoxia induces the expression of Sox2 and Oct4 genes that are related to stem cell function [12]. In particular, Sox2, together with Sox4, was recently shown to play a pivotal role in the maintenance of stemness in CSCs [13]. Under hypoxic conditions, HIF-1*α* interacts also with Notch to promote a stem cell phenotype thus supporting the role of Notch signaling on CSC stimulation mediated by hypoxia [14]. Consistently, hypoxia has been demonstrated to induce epithelial-mesenchymal transition (EMT), which prompts invasion and metastasis of cancer cells [15, 16]. EMT is a complex biologic process of epithelial cells involving cell-cell junction dissolution and loss of apicobasolateral polarity, thus promoting migratory mesenchymal properties [17]. During EMT, epithelial cells undergo several biochemical alterations that allow the acquisition of the mesenchymal phenotype enabling cancer cells to evade their “homeland” and to colonize remote locations [18]. EMT-inducers, including transforming growth factor-*β* (TGF-*β*) and hypoxia, trigger changes in gene expression by complex signaling pathways [19, 20]. An early event of EMT is the increased expression of the mesenchymal marker Vimentin and the transcriptional downregulation of E-cadherin mediated by Twist, Snail, Slug, and Zeb regulators. The downregulation of E-cadherin, a transmembrane adhesion epithelial marker involved in cell-to-cell interactions and epithelium organization [21], is related to cell junction breakdown and to signaling events that stimulate marked changes in gene expression profile. The loss of polarity and gain of motile characteristics of mesenchymal cells during embryonic development have suggested analogies with metastatic cancer cells during malignant progression [22]. Notably, recent data on several cancer types have demonstrated that EMT is involved in generating cells with properties of stem cells [23–25]. This implies that hypoxia-induced EMT may affect CSCs or induce stem-like cells from more differentiated progenitors determining an increase of CSC population responsible for early systemic cancer dissemination and metastasis formation.

**Figure 1:**
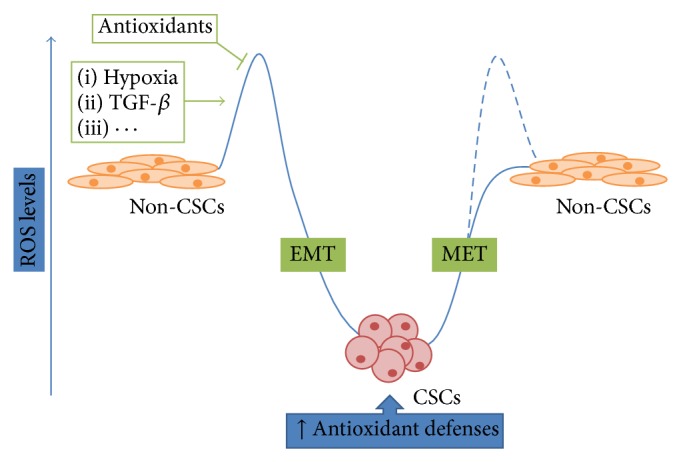
Role of ROS in the establishment and maintenance of stemness in CSCs. Changes in the redox state are highlighted in blue, while a hypothesized transitory ROS burst is indicated with dashed line. EMT and MET biological processes and their inducers/repressors are indicated in green.

Many studies have demonstrated a functional connection between low oxygen tension, ROS production, and EMT [18, 26, 27]. For instance, it has been recently discovered that activation of the NADPH oxidase family or the signaling pathway of prostate transmembrane protein androgen induced-1 (TMEPAI) may contribute to TGF-*β*-mediated EMT through ROS production in cancer cells [28–30]. However, despite the fact that ROS can favor EMT and antioxidants can attenuate hypoxia-induced EMT and metastasis dissemination in cancer cells [31], the maintenance of low ROS levels is crucial to preserve CSC self-renewal and stemness. Indeed, in contrast to cancer cells in which ROS levels are increased, CSCs generally maintain low ROS, exhibiting redox patterns that are similar to the corresponding normal stem cells [32]. Diehn et al. reported that ROS levels are lower in human and murine breast CSCs compared to non-stem breast cancer cells and that the pharmacological depletion of ROS scavengers in CSCs markedly decreases their clonogenicity and results in radiosensitization [33]. Gastrointestinal CSCs with a high level of CD44 expression have shown an enhanced capacity of reduced glutathione (GSH) synthesis and defense against ROS by activation of cystine-glutamate exchange transporter xc(−) [34]. Lin et al. have recently shown that targeting the antioxidant protein peroxiredoxin 4 (PRDX4) can amplify cell death through ROS-mediated DNA/endoplasmic reticulum damage, raising the possibility that PRDX4 may be a novel therapeutic target in glioblastoma multiforme to inhibit glioma stem cell survival and/or growth [35]. Intriguingly, Pasto et al. demonstrated that ovarian CSCs show high mitochondrial activity and are sensitive to electronic transport chain inhibitors. They discovered that total ROS levels were significantly higher in the non-CSC population than in the CSC CD44^+^/CD117^+^ subset, while mitochondrial ROS levels were significantly higher in the CD44^+^/CD117^+^ than in CD44^+^/CD117^−^ cells [36]. This apparent ambiguity was explained by hypothesizing transitory bursts of ROS production that could stimulate differentiation of CSCs towards their non-stem cancer cell counterpart. This assumption is based on the general statement that CSCs from several cancer types have redox features similar to those of normal tissue stem cells [37]. In addition, it has been observed that the hematopoietic stem cells (HSCs) fraction with high total ROS levels has higher myeloid differentiation capacity than the low ROS cell fraction, suggesting that high ROS levels may render hematopoietic stem cells “myeloid shifted,” which is one of the main features of aged HSCs [38]. Therefore, since EMT is a reversible and redox-dependent phenomenon, it is likely that ROS could also stimulate mesenchymal-epithelial transition (MET) regulating differentiation of CSCs towards non-stem cancer cells (Figure 1).

On the basis of the previously described data, it is conceivable to postulate that subsets of CSCs, similarly to normal stem cells, can regulate their differentiation and cell-cycle phase via subtle changes and fine-tuning of the redox status. In conclusion, oxidative stress caused by the cellular accumulation of ROS is intrinsically detrimental to CSCs, which have evolved antioxidant systems to protect against ROS increase. Thus, to develop rational therapies that specifically target CSCs, the clarification of their redox regulation mechanisms appears to be essential. This knowledge will allow the setting up of local and systemic oncological therapies by patient- and tumor-specific identification of CSC redox-resistance mechanisms.

## 3. Antioxidant Mechanisms and ROS-Related Markers in Cancer Stem Cells

**Figure 2:**
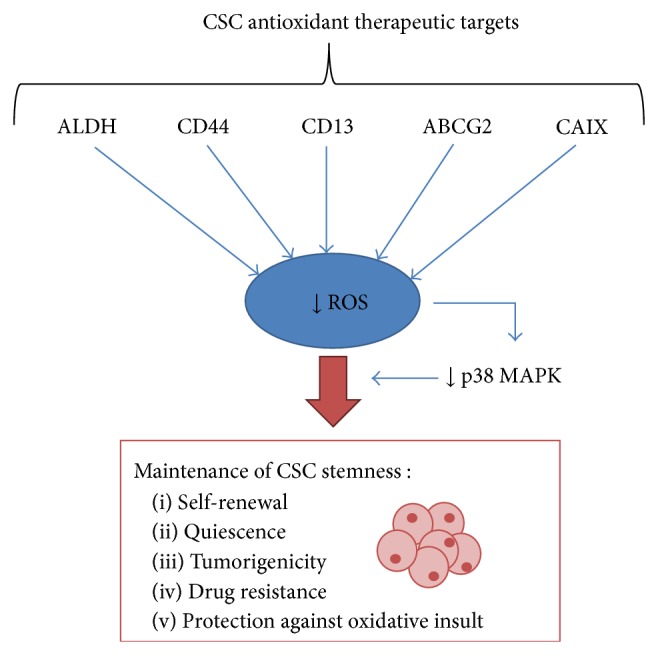
Antioxidant mechanisms and molecular markers involved in the establishment and maintenance of low ROS levels in CSCs.

In this section, the redox mechanisms that regulate stemness in tumors are reported and schematically represented in Figure 2.

### 3.1. p38 MAPK Pathway

Over the last years, mitogen-activated protein kinase (MAPK) signal transduction pathways have been shown to be involved in a wide-ranging number of biological processes, including cell proliferation, differentiation, apoptotic cell death, inflammation, and responses to various external signals [39, 40]. The most important event of p38 MAPK pathway activation is a dual phosphorylation at the Thr-Gly-Tyr motif induced in response to several stimuli including environmental and oxidative stresses, inflammatory cytokines, and TGF-*β* signaling [41]. Once activated, p38 can translocate from the cytosol to the nucleus where it phosphorylates Ser/Thr residues of many target proteins. Several studies have reported that p38 MAPK plays an important role in human tumors [42] and acts as a sensor of oxidative stress in cancer cells [43]. Sato et al. have shown that ROS-mediated stimulation of the p38 MAPK pathway controls both differentiation and tumor-initiating capacity of glioma cells. In particular, they observed that stimulation of differentiation and loss of cellular self-renewal rely on the ROS-dependent triggering of p38 pathway in glioma initiating cells, demonstrating that oxidative stress deprives these cells of their stemness property [44].

MicroRNAs, small noncoding RNA molecules that generally downregulate gene expression by base pairing with 3′ untranslated regions (3′ UTRs) of target messenger RNAs, have recently been described as important players in the modulation of CSC-related features upon modification of the redox status of the cell [45]. In particular, two members of miR-200 family (miR-141 and miR-200a), previously studied for their ability to modulate cell motility, apoptosis and stemness [46–48], were attributed with a pivotal role in redox sensing through the inhibition of p38 MAPK pathway. Indeed, Mateescu et al. have shown in tumor mouse models that the accumulation of these two miRNAs correlates with a low amount of p38*α* subunit, increased malignancy, and an oxidative stress signature [49]. Altogether, these studies strongly suggest that the downregulation of p38 MAPK signaling by miR-200 family is a common mechanism involved in the maintenance of the CSC phenotype.

### 3.2. Aldehyde Dehydrogenases

Aldehyde dehydrogenases (ALDHs) belong to a family of enzymes involved in a variety of biological processes. Among their various functions, ALDHs have been described to decrease oxidative stress caused by aldehydes, in a broad variety of normal or pathological events, including inflammation, mitochondrial respiration, and metabolism of xenobiotics [50, 51]. Indeed, aldehydes are highly reactive and relatively long-lived molecules implicated in oxidative stress-associated diseases, such as atherosclerosis, cancer, diabetes, chronic alcohol exposure, and acute lung injury, and in neurodegenerative diseases like Alzheimer and Parkinson diseases [52–54]. The ALDH superfamily includes NAD(P)+-dependent enzymes that oxidize endogenous or exogenous aldehydes to their corresponding carboxylic acids [50]. Increasing evidence has shown that high ALDH activity can be considered a general marker for CSC stemness [55], suggesting that this enzyme family may play an important role in CSC biology, including oxidative stress response, regulation of differentiation, and drug resistance. The high ALDH activity in CSCs has been mainly attributed to the isozyme ALDH1A1 and more recently to other isozymes, including ALDH1A2, ALDH1A3, ALDH1A7, ALDH2, ALDH3A1, ALDH4A1, ALDH5A1, ALDH6, and ALDH9A1 [56]. It has been demonstrated that their high activity allows CSCs to metabolize retinol to retinoic acid and thereby to modulate proliferation and differentiation. Furthermore, ALDHs can function to protect cancer cells against alkylating agents of the oxazaphosphorine (OP) family, such as cyclophosphamide (CP), one of the most efficacious anticancer agents, and its derivatives [57]. ALDHs decrease the sensitivity of the cell to the toxic effects of CP by enzyme-catalyzed bioinactivation. In addition to being OP-resistant, ALDH1A1^+^ ovarian cancer cells have also been found to be resistant to taxane and platinum treatments and to reacquire sensitivity after ALDH1A1 downregulation [58]. The detoxification capacity of the ALDHs has the potential to protect stem cells against oxidative insults and could be one of the important factors governing their longevity [55]. Recently, ALDHs have been attributed with a relevant role in stemness maintenance of breast CSCs. Wang et al. have shown that ERBB2^+^ breast cancer cells contain increased fat stores and high level of ALDH expression compared with other breast cancer cells or normal breast epithelial cells [59]. They found that the selective inhibition of PPAR-*γ*, a well-established positive regulator of adipogenesis and lipid storage, leads to a significant decrease of the ALDH^+^ cell population, specifically in ERBB2^+^ breast cancer cells [60]. Hence, these results suggest that PPAR-*γ* has a key role in maintaining CSC populations through the ALDH mediated downregulation of ROS levels. In fact, ALDH inhibition leads to accumulation of ROS to toxic levels, with the consequent DNA damage and apoptosis induction, specifically within the drug-tolerant cell subpopulation. These data reveal a potential beneficial effect of a combination therapy including ALDH inhibition to delay cancer relapse. Consistently, Raha et al. recently described a drug tolerance mechanism in cancer cell subpopulations derived from various tissues that involves the ALDH enzyme family, emphasizing a likely role for multiple ALDH family members in drug resistance [61]. Furthermore, it has been demonstrated that ALDH inhibition by disulfiram (DSF) suppresses the anchorage-independent sphere formation and reduces the number of tumor-initiating hepatocellular carcinoma (HCC) cells [62]. These effects mainly occur through the activation of ROS-p38 MAPK pathway and in part through the downregulation of Glypican 3 (GPC3), a cell surface heparan sulfate proteoglycan. In this context, our research group has shown that DSF inhibits growth of gemcitabine-resistant cancer cells through a ROS-mediated mechanism and, in combination with gemcitabine, synergistically reduces tumor mass in pancreatic cancer mice models [63]. Overall, these findings suggest that ALDHs play a key role in ROS homeostasis by maintaining low intracellular ROS levels and that DSF, a selective inhibitor of ALDH, might act as a therapeutic agent for the eradication of CSCs.

### 3.3. CD44

CD44 is a transmembrane protein involved in cellular adhesion exhibiting high affinity for hyaluronic acid (HA), a major component of the extracellular matrix [64, 65]. CD44 has been found expressed in embryonic [66], hematopoietic [67], epithelial, and cancer stem cells [68–70]. Intriguingly, among human breast cancer cell lines, the CD44^+^ cells with a high level of ALDH activity show increased tumor formation and lung colonization abilities compared to ALDH^low^/CD44^low^ cells [71], indicating a role of CD44 in the cancer metastatic process. CD44 is expressed in numerous isoforms that are generated through highly regulated alternative splicing events of its precursor mRNA [65]. Whereas the standard isoform of CD44 is mainly expressed in hematopoietic and normal epithelial cells, the CD44 isoforms (CD44v), which contain insertions in the extracellular region proximal to membrane, are highly expressed in epithelial-type carcinomas. The expression of CD44v seems to be correlated to the acquisition of CSC properties, tumor progression, and metastasis formation [72–74]. Despite the fact that the functional relevance of CD44 expression in CSCs remains to be further investigated [75], it has been shown that the knock-down of CD44 expression impacts on stem-like properties of CSC populations isolated from breast [76, 77], prostate [77], and colon cancers [69, 78], suggesting that CD44 might be a potential target for CSC-directed therapy. CD44v induced expression in colorectal CSCs has been found associated with the activation of the proto-oncoprotein c-Met [78], which promotes the invasive growth of both cancer and stem cells [79], suggesting that CD44v-mediated c-Met activation might also enhance the invasive growth potential of CSCs. A recent study has shown that spheres derived from nasopharyngeal carcinoma (NPC) cells possess CSC properties, express stemness proteins (Oct-4 and Nanog) and drug-resistant genes (MDR-1 and ABCG2), and undergo the EMT through increased CD44 expression [80]. Moreover, EMT has been shown to occur within the CD44^high^ CSC fraction [81]. Some authors have found that, in cancer cells undergoing EMT, CD44 mediates the adaption to a relatively high level of intracellular ROS, thus contributing to metastasis formation and drug resistance in tumor cells [80, 82]. Hypoxia has been found to be able to induce a strong shift of the cancer cell towards EMT leading to an increased proportion of CD44^high^ cells with consequent patterns of gene expression typical of EMT and enhanced sphere-forming ability [81]. This finding supports the crucial role of CD44 in the EMT phenotype of CSCs in NPC and in other tumors and its involvement in EMT-associated ROS production [80]. Furthermore, it has been demonstrated that, in cells expressing CD44, HA could be internalized through a caveolin-1-dependent endocytic pathway protecting both mitochondrial and nuclear DNA from oxidative damage [83, 84]. Additionally, the association of CD44 with lipid rafts and the subsequent endocytosis of HA have been shown to provide an intracellular pool of ROS-scavenging HA for decreasing mitochondrial DNA damage [85]. Alternatively, stem cells can utilize GSH as a ROS scavenger. Indeed, CD44v can interact with and stabilize the cystine transporter subunit xCT and thereby regulate the intracellular level of GSH, resulting in the suppression of p38 MAPK- and p21^CIP1/WAF1^-mediated growth inhibition of gastrointestinal and mammary CD44^+^ CSCs [34, 86].

### 3.4. CD13

CD13, also named amino peptidase N, is a member of the zinc-binding metalloproteinase superfamily and plays key roles on various cellular processes, including mitosis, invasion, cell adhesion, angiogenesis, and resistance to radiation and apoptosis [87–90]. CD13 has been recently identified as a functional marker that can be used to recognize potentially dormant and therapy-resistant liver CSCs. CD13^+^ cells were found in the G0 phase of the cell cycle and typically formed cellular clusters in cancer foci. Following treatment, these cells survive and are enriched along the fibrous capsule where liver cancer usually relapses [91]. Haraguchi et al. demonstrated that CD13^+^ cells contain low levels of ROS and show a reduced ROS-induced DNA damage after genotoxic chemo/radiation stress, which preserves cancer cells from apoptosis. Since CD13^+^ cells have a high tumorigenicity and self-renewal ability* in vivo*, the authors suggested that CD13 expression is essential for CSC protection and maintenance in the liver. Indeed, the suppression of CD13 expression inhibits both self-renewal and tumor initiation ability by a decrease in ROS production. Therefore, CD13 expression is closely related to the multidrug-resistant phenotype in slow-growing cells having a key role in the protection of cancer cells from apoptosis via ROS scavenger mechanisms [91].

### 3.5. ABCG2 Transporter

ATP-binding cassette member 2 of the subfamily G (ABCG2) is mainly expressed in side populations of various stem cells and is responsible for the maintenance of their phenotype [92]. As an important multidrug resistance transporter, ABCG2 has the capability to promote the efflux of various chemotherapy drugs contributing to cancer cell resistance [93]. Initially, ABCG2 was considered a stem cell marker in bone marrow [92] and subsequently it became a potential stemness marker in HCC [94], as it was detected in HCC and various cancer types [95–97]. In tumor tissues, ABCG2 expression correlates with high Ki67 expression, a well-established marker of cell proliferation, suggesting that ABCG2 may regulate the proliferation of tumor cells [98]. It has been hypothesized that the mechanisms underlying tumor proliferation by ABCG2 may include activation of PI3K/Akt and STAT3 signaling pathways [99, 100]. Zhang et al. have focused on the role of ABCG2 as a potential CSC marker and its modulatory effect on malignant behaviors of HCC, confirming its role in tumorigenicity, proliferation, drug resistance, migration, and metastasis formation [98]. A great number of studies have demonstrated that ABCG2 plays an important role in protecting cells from toxic agent-mediated damage and its aberrant function is linked to disease development. Moreover, other studies have shown that ABCG2 is induced by hypoxic stress as a protective mechanism to regulate toxic levels of cellular porphyrin/heme [101, 102]. Shen et al. have found that in Alzheimer disease (AD) neuronal cells expressing ABCG2 are able to (i) protect cells from ROS-induced toxicity/death; (ii) inhibit ROS-induced expression of inflammatory genes (IL-8 and GRO) and decrease ROS-induced IL-8 cytokine secretion; (iii) inhibit ROS-induced phosphorylation of I*κ*B and activation of NF-*κ*B; (iv) inhibit the uptake of hemin chloride and decrease ROS generation into the cells [103]. These findings suggest that upregulation of ABCG2 in AD brain could be involved in protecting neuronal cells from ROS-induced damage and from ROS-induced inflammatory responses via the NF-*κ*B signaling pathway. Since ABCG2 is an efflux pump located at the cellular membrane and is a well-known marker of CSCs, we can hypothesize its direct involvement in ROS homeostasis in CSCs. ABCG2 may prevent intracellular ROS level increase through its demonstrated activity of GSH transporter out of the cells. Cells overexpressing ABCG2 have indeed a high capacity to promote the efflux of reduced GSH and to increase its extracellular levels protecting cells from oxidative stress [104]. This function may be essential to regulate the redox balance of cells located in redox-sensitive regions, such as the stem cell niche. We believe that deeper studies on the role of GSH transport through ABCG2 are necessary to better understand the potential significance of this protein in regulating redox balance of progenitor cells and their ability to self-renew or differentiate.

### 3.6. Carbonic Anhydrase IX

Carbonic anhydrase IX (CAIX) is codified by one of the most strongly induced hypoxia-response genes. It is a metalloenzyme that reversibly catalyzes hydration of carbon dioxide to bicarbonate and protons, and hence it is centrally involved in the regulation of extracellular and intracellular pH [105, 106]. CAIX expression contributes to the acidification of the microenvironment, enhancing cancer cell proliferation, invasion, and metastasis formation [105, 107]. CAIX is upregulated in hypoxic tumors, where it is a marker for distant metastasis and poor survival, while depletion of CAIX expression or pharmacologic inhibition of its activity significantly constrains breast tumor growth and metastasis formation* in vivo* [108]. Given that CSCs preferentially survive in the hypoxic niche, it has been recently sought the functional involvement of CAIX in the mesenchymal and stemness phenotype regulation of breast CSCs in hypoxic conditions. Importantly, it has been shown that CAIX expression and activity are required for enrichment and functionality of breast CSCs and that CAIX is required for mTORC1 (mammalian target of rapamycin (mTOR) complex 1) signaling in hypoxia [109]. Altogether, these findings suggest that inhibition of CAIX activity may provide a way to inhibit the expansion of CSCs supporting the use of CAIX-specific inhibitors [108, 110, 111].

## 4. ROS-Related MicroRNAs in Cancer Stem Cells

**Figure 3:**
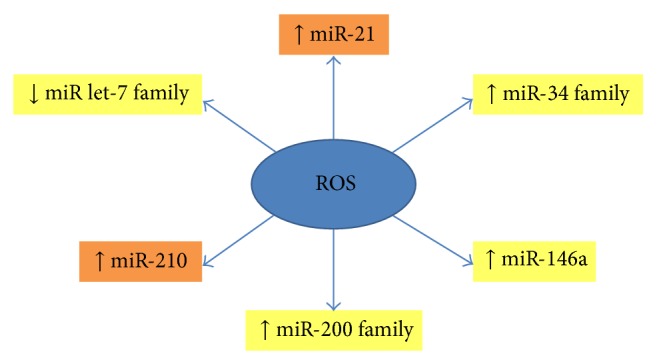
Role of ROS in microRNA regulation of CSCs. The black arrows indicate up- or downregulation of miR by ROS. The yellow box indicates high miR expression in CSCs, while the orange box indicates low miR expression in CSCs.

It has been widely recognized that microRNAs (miRNAs), a group of small non-protein-coding RNAs, act as posttranscriptional regulators of mRNAs by binding to their specific binding sites in the 3′ untranslated region (3′-UTR), resulting in either degradation of the target mRNAs or inhibition of protein synthesis [112]. Many studies demonstrated that miRNAs play a pivotal role on tumorigenesis. The altered expression of miRNA profiles has been clearly related to poor clinical outcome of tumor patients, resistance to chemotherapy, and tumor relapse. Importantly, an increasing number of miRNAs have been shown to function as regulator of CSCs and it has been associated with ROS production during tumorigenesis and tumor progression. In Figure 3 and in the following sections, we provide a description of some well-characterized miRNAs that are potentially involved in the regulation of ROS production in CSCs.

### 4.1. miR-let-7

A great amount of studies demonstrate that miR-let-7 family exerts a key regulatory role during tumorigenesis by targeting multicellular signaling pathways. In pancreatic and prostate cancer cells, several let-7 family members, such as let-7b, let-7c, and let-7d, have been displayed as negative regulators of EMT and CSC features through the modulation of PTEN and Lin28b expression, thus considering let-7 miRNAs as tumor suppressor family molecules [113–115]. Recent evidence suggests that oxidative stress decreases the expression of let-7 family in a p53-dependent manner in a variety of tumor cells [116]. Despite the fact that the molecular mechanisms underlying this regulation are not fully understood, these findings suggest that ROS may exert a pivotal role in the regulation of tumor-associated let-7 family members in CSCs.

### 4.2. miR-21

MiR-21 has a widely described oncogenic function by targeting multiple signaling pathways and by regulating a number of biological processes, as apoptosis, cell proliferation, cancer invasion, and angiogenesis [117, 118]. In several cancers types, high levels of miR-21 expression have been strongly related to poor clinical prognosis of patients [119]. Some experimental studies* in vitro* and* in vivo* in gastric and breast cancer cells revealed that the expression of miR-21 is significantly increased in CSC subpopulations, compared to non-CSC counterpart [120, 121]. Indeed, in breast CSCs the functional loss of miR-21 is able to reverse the EMT phenotype and to inhibit HIF-1, consistently with decreased capacity of cell migration and invasion [121]. Therefore, these findings strongly suggest that miR-21 plays a critical role in the regulation of CSC and EMT features and emerging evidence indicates that ROS are closely associated with increased levels of miR-21 expression in a variety of tumor cells [122, 123]. Moreover, the increased expression of miR-21 by oxidative stress is consistent with a rise in tumor cell migration and in the self-renewal capacity of prostatic and pancreatic CSCs [124, 125]. On the other side, a new study reveals that miR-21 stimulates MAPK-mediated ROS production by downregulation of superoxide dismutase enzymes (SOD2/SOD3) and sprouty homolog 2 (SPRY-2, a negative regulator of Ras-Raf-Erk signaling) leading to the promotion of tumorigenesis [126]. These findings clearly suggest that miR-21 may have a strict functional interplay with ROS during tumorigenesis.

### 4.3. miR-34

It has been documented that miR-34 family members, such as miR-34a, are underexpressed in a variety of human tumors, such as breast, ovarian, pancreatic, brain, and lung tumors [127–129]. Low levels of miR-34a, b, and c have been found to be related to poor clinical outcome of cancer patients [130, 131]. The miR-34 has been proposed to function as a tumor suppressor contributing to the inhibition of cell survival, proliferation, invasion, and metastasis formation mediated, in part, through the activation of p53 and inactivation of cyclin D1, E2F1/2, and CDK6 [132–134]. Recently, miR-34a has been found to suppress the expression of CSC signature genes, such as CD44 and EMT markers, and to attenuate tumor invasion, metastasis formation, and CSC self-renewal capacity in pancreatic and glioma stem cells [135, 136]. The interaction between ROS and miR-34 has been investigated in some* in vitro* experimental studies demonstrating that oxidative stress increases the expression of miR-34a, b, and c in a variety of cell types, such as stem, tumor, and stromal cells [137, 138]. Despite the fact that miR-34a has been found to promote renal cell senescence by inhibition of mitochondrial antioxidant enzymes [139], the comprehension of the exact mechanisms by which miR-34 family regulates ROS homeostasis during tumorigenesis needs further in-depth studies.

### 4.4. miR-146a

Decreased levels of miR-146a expression are correlated with poor clinical prognosis of prostate and pancreatic cancer patients, acting as a potent tumor suppressor [140]. Emerging data showed that miR-146a decreases NF-*κ*B activity, consistent with decreased expression of NF-*κ*B target genes, such as IL-1*β*, IL-6, IL-8, and TNF-*α* [141]. Indeed, NF-*κ*B signaling has been reported to be involved in the enrichment of CSC and EMT features by the regulation of CSC-related genes, such as Nanog, Sox2, and Lin28, as well as the EMT marker Snail [142]. Recently, it has been reported that the miR-146a expression is absent in pancreatic cancer cells while reexpression of miR-146a results in lower capacity of tumor cell invasion, consistent with inactivation of EGFR and NF-*κ*B pathways, leading to the downregulation of NF-*κ*B target genes [143]. These results suggest that the role of miR-146a in tumorigenesis and tumor progression appears to be cell lineage specific, suggesting that further investigations are needed to understand the role of miR-146a in the regulation of CSC and EMT features in the specific cancer types. Currently, only limited evidence has been published suggesting that ROS may exert a key role in tumorigenesis mediated by the regulation of miR-146a. However, it was noted in human primary monocytes that oxidative stress could induce oxidized LDLs in conjunction with increased expression levels of miR-146a and miR-146b-5p [144]. It has also been shown that metal sulfate-induced oxidative stress increases the expression of miR-146a in human astroglial cells and that the addition of antioxidant molecules inhibits miR-146a expression [145]. However, further investigations are needed to understand the exact functions of miR-146a in the regulation of ROS homeostasis during tumorigenesis and stemness.

### 4.5. miR-200 Family

miR-200 family members play very important roles in tumorigenesis by targeting multiple cellular signaling pathways and it has been shown that the miR-200 expression is decreased in a wide variety of human tumors. Moreover, miR-200 family is intimately involved in EMT and it has been associated with the acquisition of stemness and, therefore, with the formation and maintenance of tumor initiating cell-like phenotypes [146]. The alterations of miR-200 expression have been shown to be closely related to poor clinical prognosis of cancer patients [147]. It has been reported that miR-200 decreases the expression of Bmil-1, Suz12, and Notch-1, known regulators of CSC and EMT phenotypes in various cancer cells, consistent with the inhibition of CSC self-renewal capacity [148–150]. Importantly, the downregulation of miR-200a, b, and c has been observed in CSC-like (CD44^+^/CD24^−^) cells of breast cancer [48]. Overall, these data suggest that miR-200 family may work as potential tumor suppressor molecules by targeting multicellular signaling pathways. The role of miR-200 in the regulation of ROS homeostasis during tumorigenesis has not been fully elucidated. However, recent studies have identified a new function for miR-200 in the regulation of oxidative stress response. Importantly, Mateescu et al. have reported, using a microarray analysis, that the expression of the two members of miR-200s family, miR-200a and miR-141, is stimulated by oxidative stress. Mechanistically, these two miRNAs were found to target directly p38*α*, an important modulator of oxidative stress, leading to increased intracellular levels of ROS and subsequent activation of the Nrf2 oxidative stress response pathway. The increased ROS, in turn, augment expression of the miR-200s, thus establishing a miR-200s-activated stress signature, which strongly correlates with longer patient survival caused by an improved response to chemotherapeutic agents [49]. Another study demonstrated that H_2_O_2_ and other oxidant agents increase the expression of miR-200c and induce growth arrest, apoptosis, and senescence in HUVEC cells by inhibition of ZEB1 expression [151]. Overall, these findings indicate a potential role of miR-200 family in the regulation of ROS homeostasis in CSCs.

### 4.6. miR-210

Several studies have indicated that increased levels of miR-210 are related to poor clinical prognosis of breast and pancreatic cancers [152, 153]. It has been documented that hypoxia highly increases miR-210 expression, suggesting a close relationship of miR-210 with hypoxia-mediated ROS production [154]. Importantly, miR-210 is induced by NF-*κ*B, HIF-1*α*, and HIF-2*α*, activating, in turn, miR-210 promoter by HIF-1 binding to a hypoxia responsive element (HRE) on the proximal miR-210 promoter [155]. Therefore miR-210 has been proposed to play a key role in cellular adaption to hypoxia, in stem cell survival, in stemness maintenance, in the modulation of DNA damage repair pathway, and in the regulation of ROS homeostasis [156, 157]. These findings suggest that hypoxia-induced expression of miR-210 may have a pivotal role within a tumor microenvironment, further suggesting that a new therapeutic approach could be designed for the prevention and/or treatment of cancer by targeting miR-210. A preliminary study by Yang et al. found that miR-210 downregulation radiosensitizes hypoxic human hepatocarcinoma cells and suggested that miR-210 might be a potential therapeutic target to hypoxic cancer cells [158]. The same authors have subsequently demonstrated that hypoxia leads to an increased level of both HIF-2*α* mRNA expression and miR-210 expression in glioma stem cells (GSCs). In hypoxic GSCs, knock-down of miR-210 decreases neurosphere formation capacity, stem cell marker expression, and cell viability, while it induces differentiation and cell cycle G0/G1 phase arrest. Moreover, knock-down of miR-210 leads to increased apoptotic rate, stimulation of caspase-3/7 activity, and decreased invasive capacity and radioresistance. Finally, these findings suggest that miR-210 might be a potential therapeutic target to eliminate GSCs located in hypoxic niches [159].

## 5. Conclusions

The reviewed data emphasize the supporting role of hypoxia and ROS deregulation in CSC establishment and propagation. Alongside, it has now been clarified that conventional cancer therapies result in a transient reduction in tumor mass by killing non-stem cancer cells, while failing to eliminate CSCs. In addition, a general agreement exists on the formation of metastases from the dissemination of CSCs and their colonization of secondary sites. Cancer cells normally adapt to persistent oxidative stress by regulating redox response. In CSCs, such adaptation is potentiated by increased antioxidant mechanisms and ROS-related miRNA expression, thus resulting in resistance to certain anticancer agents. Thus, future improvements in cancer treatment may conceive the development of drugs that target ROS-related pathways that are specifically altered in CSCs.
